# Risk factors of stone recurrence after endoscopic retrograde cholangiopancreatography for common bile duct stones

**DOI:** 10.1097/MD.0000000000020412

**Published:** 2020-07-02

**Authors:** Peng Lujian, Cheng Xianneng, Zhang Lei

**Affiliations:** Department of General Surgery, Traditional Chinese Medicine Hospital, Chongqing, China.

**Keywords:** common bile duct stones, endoscopic retrograde cholangiopancreatography, gallstones, patient, risk factors

## Abstract

To explore the risk factors related to the recurrence of common bile duct stones (CBDS) after endoscopic retrograde cholangiopancreatography (ERCP), so as to provide reference for reducing the recurrence of CBDS after ERCP.

The clinical data of 385 patients with CBDS treated by ERCP from March 2012 to May 2016 were collected. According to the diagnostic criteria of recurrence of CBDS, the patients were divided into recurrence group and control group. The general information of the patients, personal history, past history, and surgical-related information were collected. Univariate analysis and multivariate logistic regression analysis were performed on the collected data to identify risk factors for recurrence of CBDS after ERCP.

A total of 262 patients were included in the study, of which 51 had recurrence of CBDS, with a recurrence rate of 19.46%. Multivariate Logistic analysis (Table 2) showed greasy diet (*P* = .436), history of cholecystectomy (*P* = .639) and gallstone size (*P* = .809) were not independent risk factor for recurrence of stones after ERCP in CBDS. But age ≥65 (*P* = .013), history of common bile duct incision (*P* = .001), periampullary diverticulum (*P* = .001), common bile duct diameter ≥1.5 cm (*P* = .024), ERCP ≥2 (*P* = .003), the number of stones ≥2 (*P* = .015), the common bile duct angle ≤120° (*P* = .002) and the placement of bile duct stent (*P* = .004) are important independent risk factor for recurrence of stones after ERCP in CBDS.

This study confirmed that ag ≥65, history of choledochotomy, periampullary diverticulum, diameter of common bile duct (≥15 mm), multiple ERCP, the number of stones ≥2, stent placement and angle of common bile duct < 120° were independent risk factors for recurrence of CBDS after ERCP.

## 1 Introduction

Gallstones are common and frequently occurring diseases of the digestive system, and the incidence of gallstones in China is 3% to 11%.^[1]^ In the United States, the incidence of gallstones is about 15%, of which 10% to 15% of patients are also accompanied by common bile duct stones (CBDS).^[2]^ Surgery is the best way to treat CBDS, endoscopic retrograde cholangiopancreatography (ERCP) has the advantages of simple operation, less trauma, fewer complications, and high success rate of stone removal. At present, ERCP is the first choice for the treatment of CBDS.^[3,4]^ However, ERCP has complications such as recurrence of CBDS, postoperative pancreatitis, bleeding and infection, among which the most common long-term complications are stone recurrence.^[5–7]^ At present, there are different reports on the recurrence rate of CBDS after ERCP, ranging from 4% to 24%.^[3,5,7,8]^ In addition, there are different research results on the related factors of CBDS recurrence.^[3,5,7,8]^ In general, the cause of recurrence of CBDS after ERCP is caused by many factors.^[3,5,7,8]^ How to prevent and reduce the recurrence of CBDS after ERCP has become the focus of hepatobiliary surgeons. In this study, the clinical data of ERCP patients in our hospital from March 2012 to May 2016 were collected and analyzed to explore the recurrence rate and related risk factors of ERCP in patients with CBDS, so as to provide reference for reducing the recurrence rate of CBDS after ERCP.

## 2 Materials and methods

### 2.1 Patients data inclusion and exclusion criteria

The clinical data of ERCP patients with CBDS (n = 385) from March 2012 to May 2016 were collected. Inclusion criteria:

(1) Diagnosis of CBDS by abdominal ultrasound, CT or magnetic resonance MR cholangiopancreatography;
(2) ERCP for CBDS;
(3) Patients who agreed to the study and signed informed consent;
(4) The patient can cooperate closely with the follow-up, the follow-up time is 3 years, and the follow-up data is complete.

Exclusion criteria:

(1) those with contraindications to ERCP surgery;
(2) congenital common bile duct cysts or bile duct abnormalities;
(3) bile duct tumors;
(4) duodenal papilla tumors;
(5) with intrahepatic bile duct stones;
(6) allergic to contrast agents;
(7) ERCP stone removal cannot be exhausted;
(8) those who cannot participate in the follow-up.

Diagnostic criteria for recurrence of CBDS: 6 months after complete removal of CBDS, the patient developed symptoms of biliary obstruction such as jaundice, abdominal pain, and fever. In addition, imaging examinations include color Doppler ultrasound, CT, MRCP and other imaging examinations suggesting CBDS.^[9,10]^ According to the diagnostic criteria of recurrence of CBDS, the patients included in the study were divided into recurrence group (n = 51) and control group (n = 211). All patients and their families involved in the study were informed of the study and signed the informed consent, and the study was approved by the ethics committee of Chongqing Traditional Chinese Medicine Hospital.

### 2.2 ERCP surgical procedure and postoperative management

ERCP surgical methods and procedures are reported in previous literature.^[11–13]^ ERCP was performed by the same senior surgeon. The patient was ordered to lie supine after operation, and the vital signs of the patient were closely observed. The blood amylase and blood routine of the patient were monitored 3 hours after the operation and the morning after the operation. If the patient's blood amylase has no obvious abnormality, no obvious abdominal pain and other symptoms, the patient can be asked to start eating fluid.

### 2.3 Patient follow-up

Patients were followed up for 3 years from March 2012 to June 2019. All the patients were followed up once every three months within one year. The blood biochemistry and abdominal B-ultrasonic examination were performed. After that, according to the condition of patients’ follow-up, blood biochemistry and abdominal B-ultrasonic examination were performed once every 6 months. If the blood biochemical or B-ultrasonic examination indicates the possibility of CBDS recurrence, CT and MRCP should be performed to confirm the diagnosis, and ERCP should be performed again in hospital, and follow-up should be stopped. If the patient has jaundice, abdominal pain and other symptoms, the examination of abdominal B-ultrasound, CT, MRCP and other examinations suggest that the patients with CBDS recurrence need to be hospitalized for ERCP again, and stop the follow-up.

### 2.4 Clinical data collection

Collect and record clinical data of patients included in the study:

(1) general situation: gender, age, BMI, recurrence time, etc;
(2) previous history: history of hypertension, history of diabetes, history of hepatitis B, history of intrahepatic bile duct stones, history of gallbladder stones, history of cholecystectomy, history of biliary ascaris History, history of common bile duct incision and lithotripsy;
(3) Personal history: drinking history, smoking history, history of bad habits, etc;
(4) ERCP operation: whether to perform endoscopic mechanical lithotripsy, whether to place a bile duct stent, and the number of ERCP procedures;
(5) Biliary tract and stones: presence of periampullary diverticulum, common bile duct diameter, number of stones, stone size, presence or absence of suppurative cholangitis, common bile duct stricture, bile duct angle, duodenal parapapillary diverticulum, etc.

Univariate analysis and multivariate logistic regression analysis were performed on the collected data to identify risk factors for recurrence of common bile duct stones.

### 2.5 Statistical analysis

SPSS 19.0 software was used for data analysis. Results were expressed as mean ± standard deviation (x ± s). A chi-squared test or Fisher exact test was used for the analysis of contingency tables depending on the sample size. Student's *t* test and the Wilcoxon test were used to compare clinical characteristics between recurrence group and control group. When *P* < .05, the difference is statistically significant. Multivariate logistic was used to analyze the statistical significance of the indicators and calculate the regression coefficient (β), relative odds ratio (OR) and 95% confidence interval (95% confidence interval). When *P* < .05, the difference was significant.

## 3 Result

### 3.1 Univariate analysis of postoperative recurrence of ERCP in patients with common bile duct stones

According to the exclusion and inclusion criteria, from March 2012 to May 2016, a total of 385 patients underwent ERCP treatment in our hospital due to CBDS, of which 123 were lost to follow-up and dropped out of follow-up. A total of 262 patients were included in this study. The mean age was 63.12 ± 14.1 years; there were 148 women (56.48%) and 114 men (43.51%). Mean BMI was 23.46 ± 3.75 kg/m^2^. According to the diagnostic criteria of recurrence of CBDS, 51 of 262 patients had recurrence of CBDS, the recurrence rate was 19.46%, and the average recurrence time was (20.51 ± 9.65) months. Using one-way analysis of variance (Table 1), we found that the factors affecting the recurrence of CBDS after ERCP include age, greasy diet, history of common bile duct incision, cholecystectomy, periampullary diverticulum, common bile duct diameter ≥1.5 cm, multiple stones, stone diameter ≥1.0 cm, multiple ERCP history, common bile duct angle ≤120°, bile duct stent placement. However, gender, BMI, drinking history, smoking history, history of hypertension, history of diabetes, history of hepatitis B, gallstone accompanied, biliary tract infections, and endoscopic mechanical lithotripsy are not factors for recurrence of CBDS after ERCP.

**Table 1:**
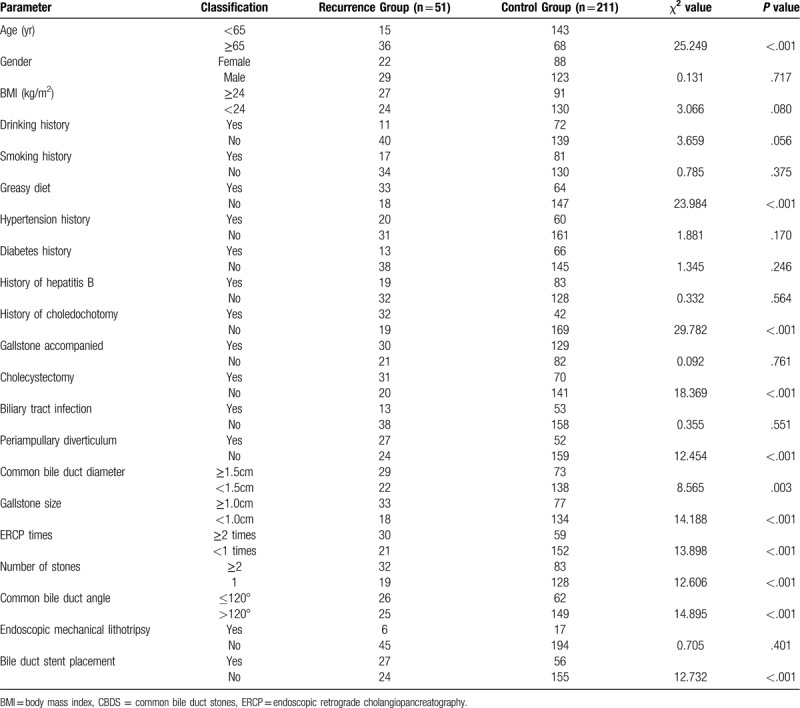
Univariate analysis of risk factors for CBDS recurrence after ERCP.

### 3.2 Multivariate logistic regression analysis of risk factors for CBDS recurrence after ERCP

Multivariate Logistic analysis (Table 2) showed greasy diet (*P* = .436), history of cholecystectomy (*P* = .639) and gallstone size (*P* = .809) were not independent risk factor for recurrence of stones after ERCP in CBDS. But age ≥ 65 (*P* = .013), history of common bile duct incision (*P* = .001), periampullary diverticulum (*P* = .001), common bile duct diameter ≥ 1.5 cm (*P* = .024), ERCP ≥ 2 (*P* = .003), the number of stones ≥2 (*P* = .015), the common bile duct angle ≤120 (*P* = .002) and the placement of bile duct stent (*P* = .004) are important independent risk factor for recurrence of stones after ERCP in CBDS.

**Table 2:**
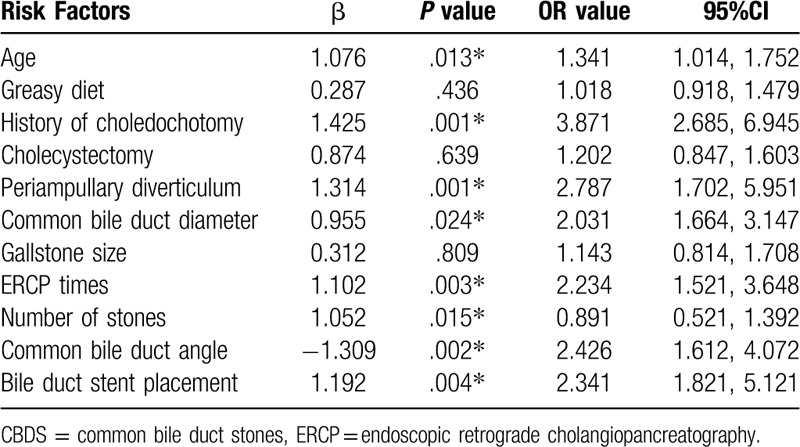
Multivariate logistic regression analysis of risk factors for CBDS recurrence after ERCP.

## 4 Discussion

In the treatment of CBDS, ERCP has a very high application value in the treatment of difficult and complex CBDS due to its characteristics of small trauma, short operation time, less complications and good prognosis. ^[3,4,14]^ However, CBDS are likely to recur within 6 months after ERCP treatment, which has become a difficult problem for clinicians. At present, there are different reports about the recurrence rate of CBDS after ERCP. The recurrence rate varies from 4% to 24%.^[3,5,7,8,15,16]^ In this study, the recurrence rate of CBDS after ERCP was 19.46%. This result indicates that the CBDS still have a high recurrence rate after ERCP treatment. Therefore, to explore the etiology and risk factors of recurrence of CBDS after ERCP is an important measure to reduce the recurrence of stones after ERCP.

At present, there are different results and conclusions on the relationship between age and recurrence of CBDS after ERCP. Some scholars think that age is the recurrence factor of CBDS after ERCP. Others think that age is not related to the recurrence of CBDS after ERCP.^[2,17,18]^ In this study, we found that age over 65 was an independent risk factor for recurrence of CBDS after ERCP. The results may be related to the lack of physical activity and greasy diet in elderly patients, as well as decreased duodenal papillary sphincter function with age, bile duct wall tension and insufficient bile duct motility, and poor bile drainage. Previous studies have suggested that cholecystectomy is a risk factor for the recurrence of CBDS after ERCP.^[19,20]^ In this study, we found that cholecystectomy was not a risk factor for recurrence of CBDS after ERCP. But, however, the history of common bile duct incision is an independent risk factor for the recurrence of CBDS after ERCP. The possible reasons are as follows:

(1) bile duct suture and injury after common bile duct incision can lead to bile duct stricture, and the residual foreign body can be used as the core to promote the formation of stones;
(2) The common bile duct bending angle after common bile duct incision is an anatomical risk factor for recurrence of stones after endoscopic common bile duct lithotripsy.

Previous studies have suggested that multiple ERCP treatment and multiple stones (≥2) are risk factor for the recurrence of CBDS after ERCP.^[21–24]^ In this study, we found that multiple ERCP (≥2) treatment and multiple stones (≥2) are independent risk factor for the recurrence of CBDS after ERCP. However, stone size ≥1 cm is not a risk factor for recurrence of choledocholithiasis after ERCP. The possible reason is that multiple ERCP and multiple calculi mechanically damage the duodenal sphincter, resulting in dysfunction of the duodenal sphincter. Eventually, the pressure in the bile duct drops, and intestinal bacteria and intestinal contents easily return to the common bile duct, which eventually leads to recurrence of stones.

Studies suggest that common bile duct diameter ≥15 mm compared with ≤10 mm, the recurrence rate of CBDS after ERCP is 19.5% and 4.9%, respectively, and the common bile duct diameter ≥15 mm is the risk factor of recurrence of CBDS after ERCP.^[25,26]^ In this study, we also found that the diameter of common bile duct ≥15 mm is a high risk factor for recurrence of CBDS after ERCP. The possible reason is that the dilated bile duct has a dysfunction, which results in poor bile drainage and cholestasis. At the same time, secondary bacterial infections also provide the core for stone formation. The prevalence of periampullary diverticulum in the general population is about 20%. The existence of periampullary diverticulum is related to the recurrence of CBDS after ERCP.^[27]^ In this study, we found that periampullary diverticulum is a high risk factor for recurrence of choledocholithiasis after ERCP, which may be related to mechanical compression of periampullary diverticulum and secondary infection of residual food in the diverticulum, resulting in biliary obstruction and accelerating the formation of choledocholithiasis. Yoo et al suggested that the common bile duct angle (<135°) was related to the recurrence of CBDS after ERCP.^[19]^ Zhang et al found that the angle of the end bile duct (<135°) was an independent risk factor for stone recurrence after ERCP.^[28]^ In this study, we found that the angle of common bile duct less than 120° is an independent risk factor for stone recurrence after ERCP. The possible reason is that the angle of common bile duct can lead to cholestasis, bile discharge is not smooth, and promote stone formation and recurrence. At present, there are few reports on the relationship between biliary stent placement and recurrence of CBDS after ERCP. In this study, we found that biliary stent placement is an independent risk factor for recurrence of CBDS after ERCP. The possible causes of recurrence of choledocholithiasis are as follows: first, the bile salt is easy to deposit and attach to the stent after choledocholithiasis, which can be used as the primary focus of choledocholithiasis; the second is that after stent placement, it will affect the dynamics of the bile duct and easily lead to cholestasis and concentration. The concentrated bile can stimulate the inflammatory changes of the bile duct mucosa, and make the leukocytes, fibrin and exfoliated epithelial cells more easily to be separated out. In addition, concentrated bile can cause increased concentrations of unbound bilirubin, calcium ions, glycoproteins, and bile acids, and promote the formation of bile pigment stones. Although there are some findings in this study, there are still some limitations in this study. First, due to the characteristics of surgical proficiency and population differences, there may be inherent errors in the results of this study. Second, the sample size of this study is too small to fully represent the actual situation of all cases. Third, this study has all limitations and risk of bias inherent to study design.

## 5 Conclusion

In summary, age ≥65, history of common bile duct incision, diverticulum around ampulla, common bile duct diameter (≥15 mm), multiple ERCPs, number of stones ≥2, bile duct stent placement and common bile duct angle 120° is a higher risk of recurrence of CBDS in patients with CBDS after ERCP.

## Author contributions

**Conceptualization:** Peng Lujian, Zhang Lei.

**Data curation:** Peng Lujian, Cheng xianneng, Zhang Lei.

**Formal analysis:** Peng Lujian, Cheng xianneng, Zhang Lei.

**Methodology:** Peng Lujian, Cheng xianneng, Zhang Lei.

**Writing – original draft:** Peng Lujian, Zhang Lei.

**Writing – review & editing:** Peng Lujian, Zhang Lei.
